# 18ß-glycyrrhetinic acid derivative promotes proliferation, migration and aquaporin-3 expression in human dermal fibroblasts

**DOI:** 10.1371/journal.pone.0182981

**Published:** 2017-08-16

**Authors:** Chi-Feng Hung, Chien-Yu Hsiao, Wen-Hao Hsieh, Hsin-Ju Li, Yi-Ju Tsai, Chun-Nan Lin, Hsun-Hsien Chang, Nan-Lin Wu

**Affiliations:** 1 School of Medicine, Fu Jen Catholic University, New Taipei City, Taiwan; 2 Department of Fragrance and Cosmetic Science, Kaohsiung Medical University, Kaohsiung, Taiwan; 3 Graduate Institute of Biomedical and Pharmaceutical Science, Fu Jen Catholic University, New Taipei City, Taiwan; 4 Department of Nutrition and Health Sciences, Chang Gung University of Science and Technology, Kweishan, Taoyuan, Taiwan; 5 Department of Chemistry, Fu Jen University, New Taipei City, Taiwan; 6 College of Pharmacy, Kaohsiung Medical University, Kaohsiung, Taiwan; 7 Children's Hospital Informatics Program, Harvard-Massachusetts Institute of Technology/Division of Health Sciences and Technology, Harvard Medical School, Boston, Massachusetts, United States of America; 8 Department of Medicine, Mackay Medical College, New Taipei City, Taiwan; 9 Department of Dermatology, MacKay Memorial Hospital, Taipei, Taiwan; 10 Mackay Junior College of Medicine, Nursing, and Management, New Taipei City, Taiwan; Universidade do Porto, Faculdade de Farmácia, PORTUGAL

## Abstract

Licorice (*Glycyrrhiza*) species have been widely used as a traditional medicine and a natural sweetener in foods. The 18β-glycyrrhetinic acid (18β-GA) is a bioactive compound in licorice that exhibits potential anti-cancer, anti-inflammatory, and anti-microbial activities. Many synthesized derivatives of 18β-GA have been reported to be cytotoxic and suggested for the treatment of malignant diseases. In this study, we explored the possible pharmacological roles of an 18β-GA derivative in skin biology using primary human dermal fibroblasts and HaCaT keratinocytes as cell models. We found that this 18β-GA derivative did not cause cell death, but significantly enhanced the proliferation of dermal fibroblasts and HaCaT keratinocytes. A scratch wound healing assay revealed that the 18β-GA derivative promoted the migration of fibroblasts. Due to the important role of aquaporin-3 in cell migration and proliferation, we also investigated the expression of aquaporin-3 and found this compound up-regulated the expression of aquaporin-3 in dermal fibroblasts and HaCaT keratinocytes. In dermal fibroblasts, the 18β-GA derivative induced the phosphorylation of Akt, ERK, and p38. The inhibitor of Akt predominantly suppressed the 18β-GA derivative-induced expression of aquaporin-3. Collectively, this compound had a positive effect on the proliferation, migration, and aquaporin-3 expression of skin cells, implying its potential role in the treatment of skin diseases characterized by impaired wound healing or dermal defects.

## Introduction

The roots and rhizomes of licorice (*Glycyrrhiza*) species have been widely used in herbal medicine for a long time. The genus *Glycyrrhiza* (Leguminosae) contains many species and is also known as liquorice, kanzoh, gancao, sweet root, or yasti-madhu [1]. Many experimental and clinical studies on licorice have demonstrated its potential pharmacological properties, including anti-viral, anti-microbial, anti-inflammatory, anti-oxidative, anti-diabetic, anti-asthma, and anti-cancer activities, in different organ systems [2]. Licorice has also been used in a number of foods because of its sweet flavor [3], and as an ingredient in cosmetic products to attenuate inflammation and hyperpigmentation [4, 5].

The active components derived from *Glycyrrhiza* species include saponins, flavonoids, isoflavones, coumarins, and stilbenoids [1]. Glycyrrhizic acid (glycyrrhizin), a triterpenoid saponin glycoside, is the major water-soluble constituent of licorice [3, 6]. Glycyrrhetinic acid is another natural triterpenoid in licorice, which is obtained by hydrolyzing glycyrrhizic acid by glucuronidase; it exists in two forms: 18α-glycyrrhetinic acid (18α-GA) and a stereoisomeric form, 18β-glycyrrhetinic acid (18β-GA) [6]. Multiple biological functions have been attributed to 18β-GA, including immunomodulation, anti-inflammatory effects, anti-oxidant responses, and anti-carcinogenetic activity [3]. 18β-GA is a triterpene with two functional groups (COOH and OH) in the structure and can be obtained cheaply and easily. Thus, many derivatives of 18β-GA have been synthesized, based on its structure [7]. Most of these derivatives are cytotoxic, and have been evaluated for possible anti-tumor properties [8]. Some 18β-GA derivatives have served as anti-inflammatory and antioxidant agents [9]. Different 18β-GA derivatives have also been reported to have inhibitory effects on tyrosinase activity [10].

Well-coordinated proliferation and migration of keratinocytes and fibroblasts is critical for the process of wound repair [11]. Keratinocytes migrate towards the wound gap in the early stages, and the proliferation and migration of fibroblasts happens in the subsequent stages [12]. The dermal fibroblasts then actively and dynamically migrate to the wound site and express important mediators and extracellular components, which is considered a fundamental step in wound healing [13–15]. In addition, the biological activity of fibroblasts is important in the skin aging process. Replicative senescence and functional alterations in dermal fibroblasts are critical pathogenic factors, and several cosmetic treatments that improve aged skin can stimulate a robust wound healing response by modulating dermal fibroblasts and dermal extracellular matrix [16].

Aquaporins (AQPs), also called aquaglyceroporins, are membrane proteins that transport small uncharged molecules such as glycerol, urea, and water [17]. AQPs are widely distributed in fluid-transporting tissues such as kidneys and non-fluid-transporting tissues such as skin epidermis, astroglia, and fat tissue [18]. Among the different types of AQPs, AQP-3 is the most abundant, as well as the most well-studied and validated one [19, 20]. AQP-3-knockout mice have impaired stratum corneum hydration, compared to wild-type mice [21]. In addition, delayed wound healing process with reduced keratinocyte proliferation and migration was also observed in AQP-3-knockout mice [22]. Furthermore, AQP-3 has been reported to regulate human skin fibroblast migration, indicating its role in the wound healing process [23]. These findings indicated that AQP-3 played a critical role in regulating the proliferation and migration of skin cells.

Previous studies have demonstrated that the 18β-GA derivative (18β-GA-d) used in this study had significant inhibitory effects on the formation of superoxide anions in rat neutrophils stimulated by formyl-Met-Leu-Phe/cytochalasin B (fMLP/CB) or 12-O-tetradecanoylphorbol-13-acetate (TPA) [9]. In this study, we explored other possible effects of this 18β-GA-d on the biological functions of the skin using primary human dermal fibroblasts derived from adult foreskin and HaCaT keratinocytes as cell models. We first investigated the effects of this 18β-GA-d on cell proliferation and migration, which are critical for skin homeostasis. Furthermore, because AQP-3 is the predominant skin AQP crucial for the physiological functions of the skin such as cell proliferation, migration, and intact barrier function, we also evaluated the influence of the 18β-GA-d on the expression of AQP-3 in skin cells.

## Materials and methods

### Reagents

Aprotinin, 3-(4,5-dimethylthiazol-2-yl)-2,5-diphenyltetrazolium bromide (MTT), leupeptin, phenylmethylsulfonyl fluoride (PMSF), sodium fluoride (NaF), propidium iodide, sodium orthovanadate, mitomycin C, type I collagen, and tetrahydrofuran (THF) were purchased from Sigma Chemical Co. (St Louis, MO). Antibodies (Abs) against AQP-3 (sc-20811), p38 (sc-535), ERK2 (sc-154), JNK1/3 (sc-474) were obtained from Santa Cruz Biotechnology (Santa Cruz, CA). Abs against p-AKT (#9271), Akt (#9272), p-ERK1/2 (#9101), p-P38 (#9211), p-JNK (#9251), and α-tubulin (#2125) were obtained from Cell Signaling Technology (Beverely, MA). U0126, MK2206, and SB203580 were obtained from Merck Millipore (Billerica, MA). The 18β-GA derivative (18β-GA-d) was provided by Dr. Chun-Nan Lin. For all the experiments in the study, the 18β-GA-d was dissolved in THF.

### Cell culture

Primary human dermal fibroblasts were isolated from adult foreskins. The foreskin samples were collected from 8 donors. For each experiment, at least 3 independent cell samples were collected. Briefly, the skin was divided and incubated in 1% protease (Sigma-Aldrich, St Louis, MO) overnight at 4°C to separate the dermis from the epidermis. The dermal parts were collected and cut into small pieces, and then maintained in a Dulbecco’s modified Eagle’s medium (DMEM) with 10% fetal calf serum (GibcoBRL, Invitrogen Life Technologies, Carlsbad, CA), 100 units/mL penicillin, and 100 μg/mL streptomycin (Sigma, St Louis, MO). The dermal fibroblasts that migrated from the dermal tissue were collected and subcultured. The experiments were conducted according to the principles of the Declaration of Helsinki, and were approved by the ethics committee of the Mackay Memorial Hospital. Written informed consent was obtained from each donor before the experiments.

Human immortalized keratinocytes (HaCaT cells) [24] were maintained in DMEM with 10% fetal calf serum (GibcoBRL, Invitrogen Life Technologies, Carlsbad, CA), 100 units/mL penicillin, and 100 μg/mL streptomycin (Sigma, St Louis, MO).

### Cell viability assay

For the MTT assay, cells were plated in 24-well plates (30000 cells/well). Briefly, MTT (0.5 mg/ml in the media) was used for quantifying the metabolically active vial cells. Mitochondrial dehydrogenases metabolized MTT into a purple formazan dye, which was measured photometrically at 550 nm. Cell viability was proportional to the absorbance measured. For the trypan blue exclusion assay, cells were plated in 6-well plates (100000 cells/well). After the indicated treatments, the cells were trypsinized by 0.5% trypsin-EDTA (GibcoBRL, Invitrogen Life Technologies, Carlsbad, CA) and centrifuged at 1100 rpm. The cell pellet was disintegrated in the culture medium and stained with trypan blue for enumerating the vial cells [25].

### Western blot

The procedure was performed, as described in a previous study [25]. Briefly, cells were lysed in a radioimmunoprecipitation assay buffer and centrifuged after sonication, after the indicated treatments. The protein content was quantified using a Pierce protein assay kit (Pierce, Rockford, IL), and total protein was evaluated by performing electrophoresis on 8% SDS–polyacrylamide gels and then electroblotting the separated proteins onto PVDF membranes. Specific antibodies were added, and immunoblots were detected using enhanced chemiluminescence (Chemiluminescence Reagent Plus from NEN, Boston, MA). The results were quantified by Image J software (National Institutes of Health, USA).

### Quantitative real-time PCR

The cells were lysed after the indicated treatments, and the total RNA was extracted using the TRIzol® Reagent (Thermo Fisher Scientific, Waltham, MA). First strand cDNAs were synthesized using the HiScript I TM First Strand cDNA Synthesis Kit (BIONOVAS Biotechnology, Toronto, Ontario, Canada), according to the manufacturer’s instructions. PCR was performed in 96-well plates using the KAPA SYBR® FAST qPCR KITS (Kapa Biosystems, Wilmington, MA) and determined using the CFX96™ Real-Time PCR Detection System (Bio-Rad, Hercules, CA). The expression level of the AQP-3 mRNA was analyzed by normalizing with the housekeeping gene, β-actin. The specific primers for human AQP-3 were: forward primer, 5′-GACAGAAGGAGCTGGTGTCC-3′, and reverse primer, 5′-ATGAGGATGCCCAGAGTGAC-3′ [26]. The specific primers for β-actin were: forward primer, 5′-CGGGGACCTGACTGACTACC-3′, and reverse primer, 5′-AGGAAGGCTGGAAGAGTGC-3′ [27].

### *In vitro* scratch wound healing assay

To evaluate the motility of the cultured primary human dermal fibroblasts, we used the scratch test procedure, as previously described [28]. Briefly, cells were incubated in a serum-free DMEM for 24 h, and wounds were introduced in confluent monolayers of the fibroblasts. The medium was then replaced with a serum-free medium containing 0.1% THF (control vehicle) or 3 μM, 10 μM, or 30 μM of the 18β-GA-d. The migratory condition was photographed at 6, 12, and 24 h after replacing the medium.

### Electric Cell-substrate Impedance Sensing (ECIS^TM^)

The ECIS Model 1600R (Applied BioPhysics, Troy, NY) was used to assay the migration of human dermal fibroblasts after treatment with THF or 18β-GA-d, using the procedure described previously [28]. Briefly, each ECIS well was coated with type I collagen and filled with a serum-free medium. A fibroblast suspension was then seeded into each well. An AC current shock was delivered through small active electrodes in the central part of each well to inflict the wounds. The medium was replaced with a new medium containing THF or 18β-GA-d. The wounded area in each well was gradually healed by the migration of the viable cells. The migratory response was measured in real-time by recording the recovery of electrical impedance.

### Statistical analysis

Data were expressed as the mean ± SEM of at least three independent experiments. One-way ANOVA with Tukey’s post hoc test was performed. *P* < 0.05 was considered indicative of statistical significance. All statistical analyses were conducted using the SPSS ver. 21.0 software program (IBM, Armonk, NY).

## Result

### Growth of human dermal fibroblasts was increased by the 18β-GA-d

**Fig 1:**
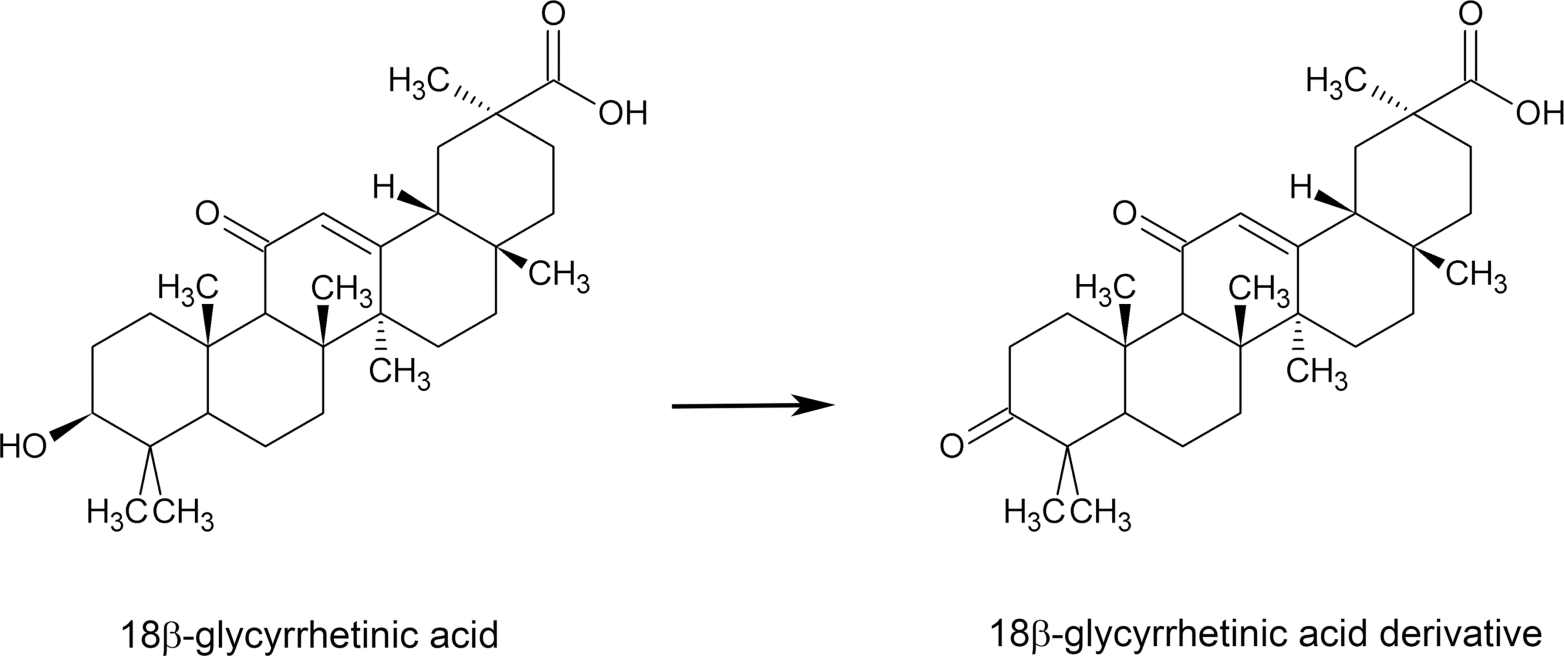
The chemical structure of the 18β-glycyrrhetinic acid derivative. The chemical structure of the 18β-glycyrrhetinic acid derivative (18β-GA-d), and the differences between 18β-GA-d and 18β-GA.

**Fig 2:**
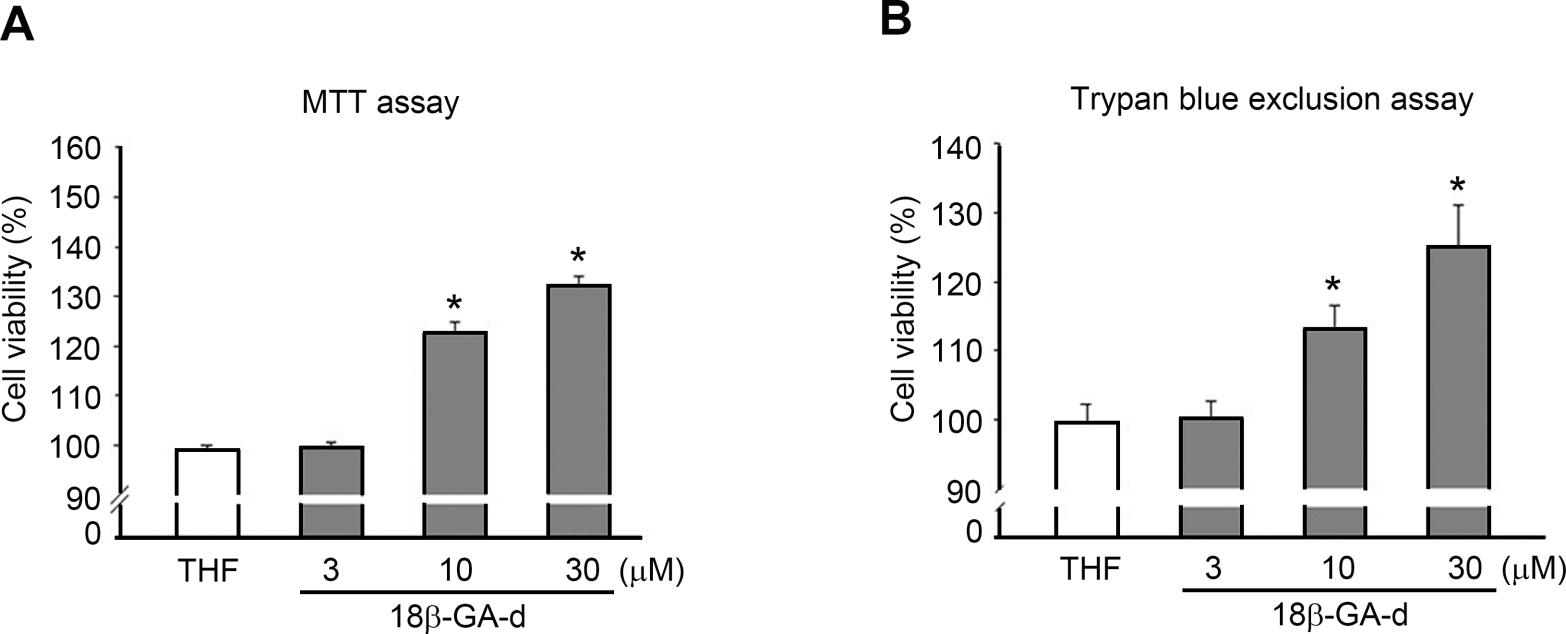
18β-GA-d increased the proliferation of human skin fibroblasts. Human dermal fibroblasts were treated with 0.1% THF (control) or different doses of 18β-GA-d (3, 10, 30 μM) for 24 h. Cell proliferation was analyzed by MTT assay (A) and by trypan blue exclusion assay (B). *p < 0.05 (mean ± S.E.M. n = 3).

As shown in Fig 1, the 18β-GA-d was derived from the chemical structure of 18β-GA after a modification at the C3 position. To explore the potential effects of this 18β-GA-d on skin, we used primary human dermal fibroblasts derived from adult skin as a cell model. Human dermal fibroblasts were treated with the 18β-GA-d for 24 h. The MTT assay revealed that the relative cell number increased in a dose-dependent manner (Fig 2A). To confirm this observation, we used the trypan blue exclusion assay to count the viable cells, and found a significant enhancement in cell growth after treatment with the 18β-GA-d. These results implied that the 18β-GA-d was not toxic for human dermal fibroblasts at these concentrations, and that it promoted the growth of human dermal fibroblasts.

### 18β-GA-d promoted the migration of human dermal fibroblasts

**Fig 3:**
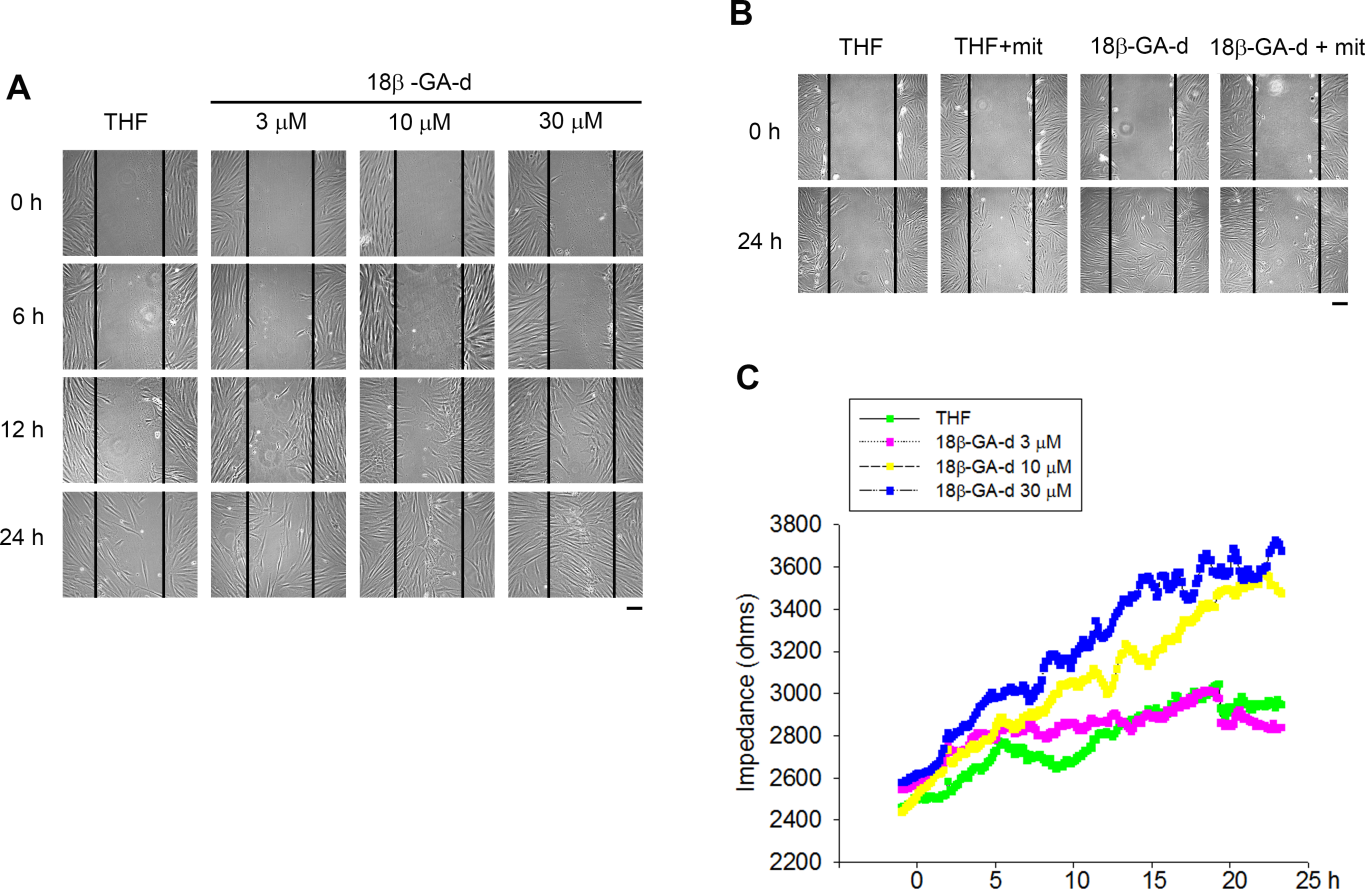
18β-GA-d increased the migration of human skin fibroblasts. (A) Human dermal fibroblasts were treated with 0.1% THF or different doses of 18β-GA-d (3, 10, and 30 μM) and observed at different times (6, 12, and 24 h). *In vitro* scratch wound healing assay was performed. Cell migration was analyzed using a phase-contrast microscope. Scale bar = 200 μm. (B) Human dermal fibroblasts were treated with mitomycin C (mit) (10 μg/ml) and 18β-GA-d (30 μM), and observed 24 h after treatment. Scale bar = 200 μm. (C) Human dermal fibroblasts on the wells with Electric Cell-substrate Impedance Sensing (ECIS) microelectrodes were wounded by a high electrical voltage. The medium was replaced with a new medium containing 0.1% THF or different doses of 18β-GA-d (3, 10, and 30 μM), and the cell migration was determined by ECIS. The migration of fibroblasts to the wounded area by electrodes was assayed using real-time measurement of the electrical impedance.

To evaluate other potential effects of 18β-GA-d on human dermal fibroblasts, we used an *in vitro* scratch wound healing assay to investigate the effect of the 18β-GA-d on the motility of human dermal fibroblasts. As shown in Fig 3A, we applied different doses of the 18β-GA-d and observed the migratory condition at different times. The wound was healed more rapidly in the group treated with the 18β-GA-d than in the control group. However, as the 18β-GA-d could promote the growth of human dermal fibroblasts, it might affect the evaluation of the scratch assay; therefore, we used mitomycin C to inhibit cell growth. Even after treatment with mitomycin C, the wound area was lesser in the group treated with the 18β-GA-d than in the control group (Fig 3B). These results indicated that the 18β-GA-d could improve the migratory ability of cells through a mechanism not associated with the increase in cell numbers. Furthermore, we used Electric Cell-substrate Impedance Sensing (ECIS) to investigate the dynamic changes in cell migration. As shown in Fig 3C, 10 and 30 μM of the 18β-GA-d increased the impedance over time, indicating the enhancement of cell migration after treatment with the 18β-GA-d.

### Up-regulated expression of AQP-3 in human dermal fibroblasts induced by the 18β-GA-d

**Fig 4:**
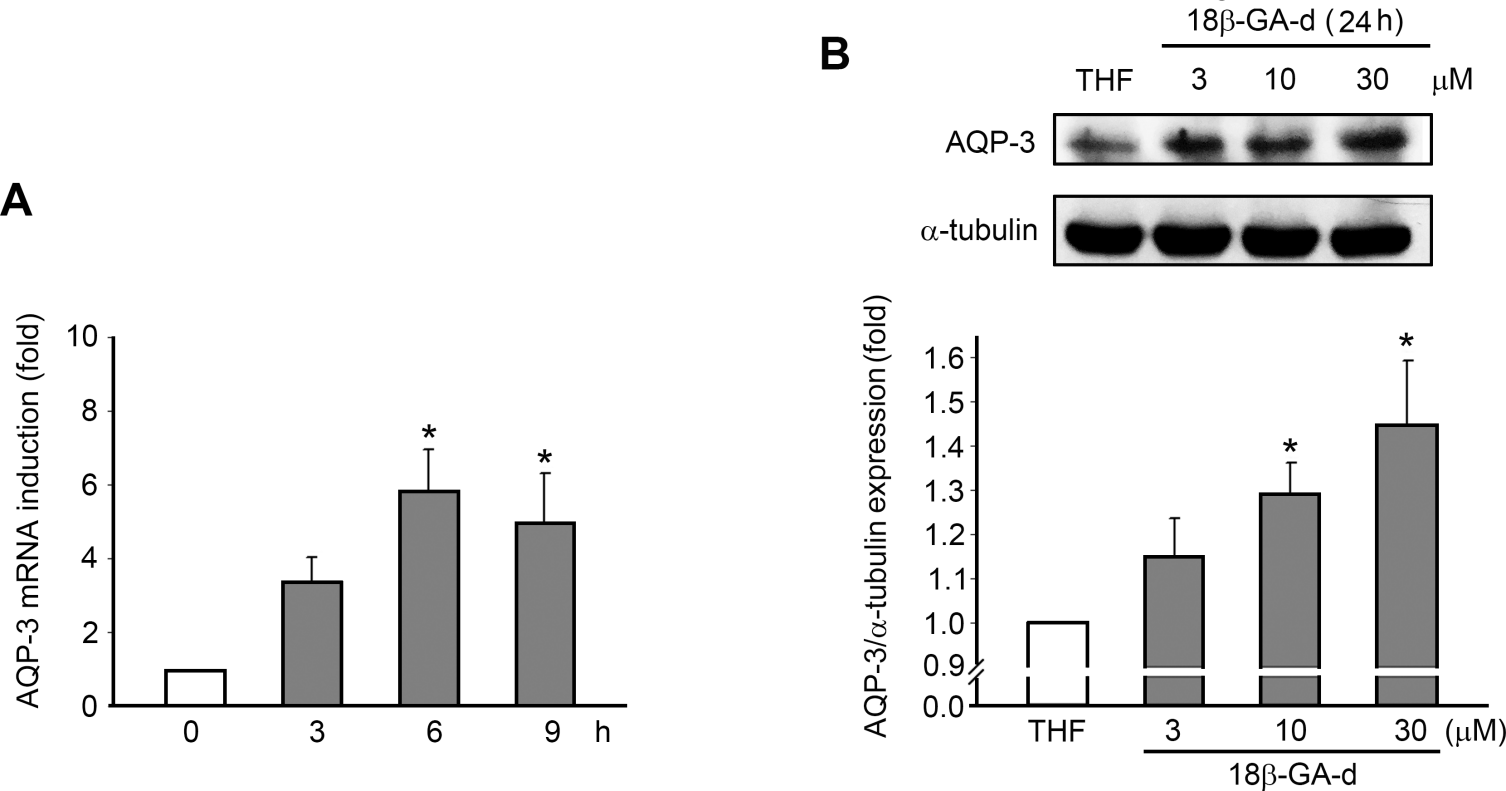
18β-GA-d induced AQP-3 expression in human dermal fibroblasts. (A) Human dermal fibroblasts were treated with 0.1% THF or 18β-GA-d (30 μM) and harvested at different times (3, 6, 9 h). AQP-3 mRNA expression, relative to THF, at each time point, was analyzed by quantitative real-time PCR. (B) Human dermal fibroblasts were treated with 0.1% THF or different doses of 18β-GA-d (3, 10, 30 μM) for 24 h. AQP-3 protein expression was analyzed by western blot assay. The quantification data for ratios of AQP-3/α-tubulin in the 18β-GA-d-treated group relative to AQP-3/α-tubulin in the THF-treated group are shown in the lower panel. *p < 0.05 (mean ± S.E.M. n = 3).

Because of the well-known roles of AQP-3 in cell growth and skin wound healing [22], we investigated the effect of the 18β-GA-d on the expression of AQP-3 in human dermal fibroblasts. We evaluated the expression of the AQP-3 mRNA in human dermal fibroblasts through quantitative real-time PCR. The results showed that the 18β-GA-d significantly induced the expression of the AQP-3 mRNA with optimal effects after 6 h (Fig 4 A). Regarding protein expression, we found that 30 μM of the 18β-GA-d up-regulated the expression of AQP-3 in human dermal fibroblasts, while 3 μM of the 18β-GA-d did not efficiently induce AQP-3 expression (Fig 4B).

### Signaling induced by the 18β-GA-d in human dermal fibroblasts

**Fig 5:**
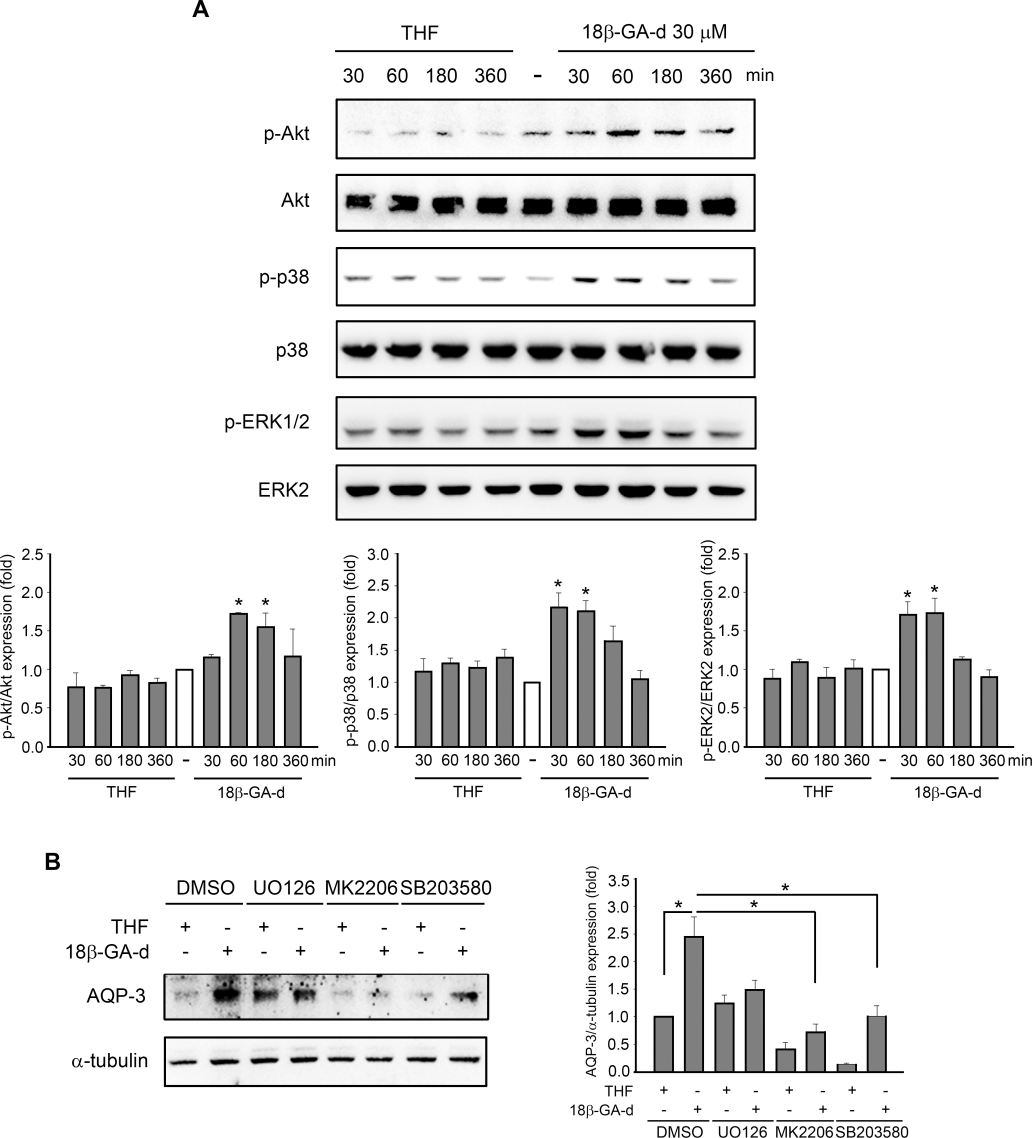
18β-GA-d increased the phosphorylation of Akt, ERK1/2, and p38 in human dermal fibroblasts. (A) Human dermal fibroblasts were treated with 0.1% THF or 18β-GA-d (30 μM) for different times. The level of phosphorylation of Akt, ERK1/2, and p38 at different times was analyzed by western blot assay. The quantification data for the ratios of phosphorylated protein/total non-phosphorylated protein in THF- and 18β-GA-d- treated groups to those in the untreated groups are shown in the lower panels. *p < 0.05 compared with THF treatment at the same time point (mean ± S.E.M. n = 3). (B) Human dermal fibroblasts were pretreated with UO126 (ERK inhibitor) (20 μM), MK2206 (Akt inhibitor) (5 μM), or SB203580 (p38 inhibitor) (10 μM) and then treated with 0.1% THF or 18β-GA-d (30μM) for 24 h. The protein expression of AQP-3 was evaluated by western blot assay. The quantification data for ratios of AQP-3/α-tubulin in different groups, relative to AQP-3/α-tubulin in the THF-treated group, are shown in the right panel. *p < 0.05 (mean ± S.E.M. n = 3).

Previous reports have indicated the involvement of Akt, ERK, and p38 signaling pathways in the migration and proliferation of fibroblasts; these pathways are also important for regulating the expression of AQP-3 induced by different stimuli [23, 29, 30]. Therefore, we investigated the effect of the 18β-GA-d on the activation of these related signaling molecules in human dermal fibroblasts. As shown in Fig 5A, treatment with the 18β-GA-d could gradually induce the phosphorylation of Akt, ERK1/2, and p38, compared to treatment with the control vehicle THF. The phosphorylation of JNK, induced by THF or the 18β-GA-d, was barely detected (data not shown). These results indicated that the Akt, ERK1/2, and p38 pathways could possibly regulate the pharmacological effects of the 18β-GA-d on human dermal fibroblasts. We then studied the possible pathways that regulated the 18β-GA-d-induced expression of AQP-3. The pharmacological inhibitors of ERK, p38, and Akt were applied. We found that Akt and p38 inhibitors significantly reduced the AQP-3 expression induced by 18β-GA-d, and Akt inhibitor predominantly suppressed the AQP-3 induction, suggesting the critical role of Akt activation in the regulation of the 18β-GA-d-induced AQP-3 expression.

### The 18β-GA-d enhanced the proliferation and migration of HaCaT keratinocytes

**Fig 6:**
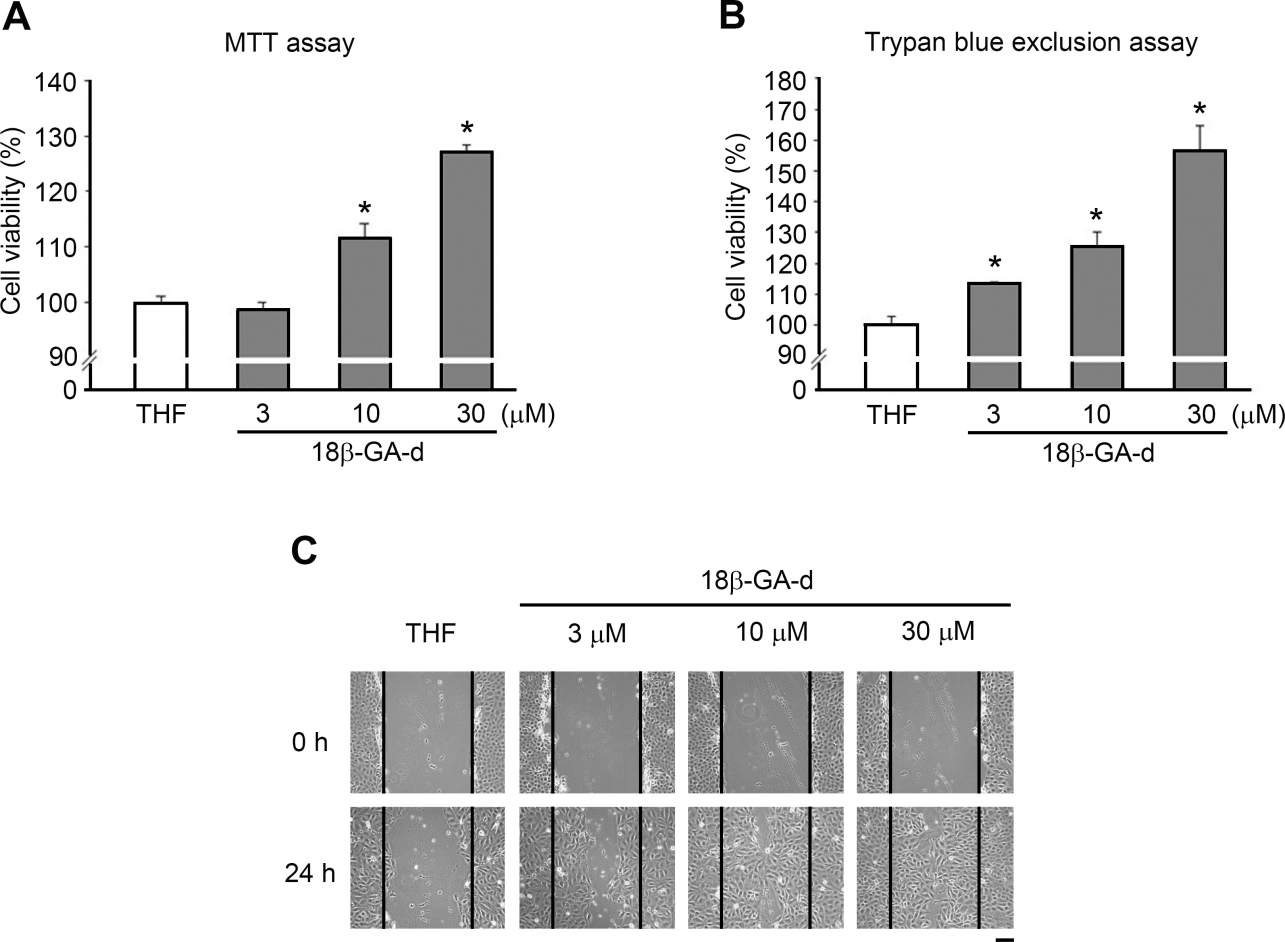
18β-GA-d increased cell proliferation in HaCaT keratinocytes. (A) HaCaT keratinocytes were treated with 0.1% THF or different doses of 18β-GA-d (3, 10, and 30 μM) for 24 h. The relative cell number was analyzed by MTT assay. (B) Similar to (A), relative cell number was determined using the trypan blue exclusion assay. (C) HaCaT keratinocytes were treated with 0.1% THF or different doses of 18β-GA-d (3, 10, and 30 μM), and an *in vitro* scratch wound healing assay was performed. Cell migration was observed at different times (0 and 24 h). Scale bar = 200 μm. *p < 0.05 (mean ± S.E.M. n = 3).

We next investigated whether the 18β-GA-d had similar effects on keratinocytes. We evaluated the growth and migration of a human keratinocyte cell line, HaCaT, after treatment with the 18β-GA-d. After treating HaCaT keratinocytes with the 18β-GA-d at different concentrations, we observed a significant increase in cell number, as measured by the MTT assay; an especially prominent effect was exerted by 30 μM of the 18β-GA-d (Fig 6A). The trypan blue exclusion assay confirmed the effect of the 18β-GA-d on HaCaT (Fig 6B). Furthermore, we performed an *in vitro* scratch wound healing assay and showed that the 18β-GA-d could promote the motility of HaCaT keratinocytes (Fig 6C).

### The 18 β-GA-d induced AQP-3 expression in HaCaT keratinocytes

**Fig 7:**
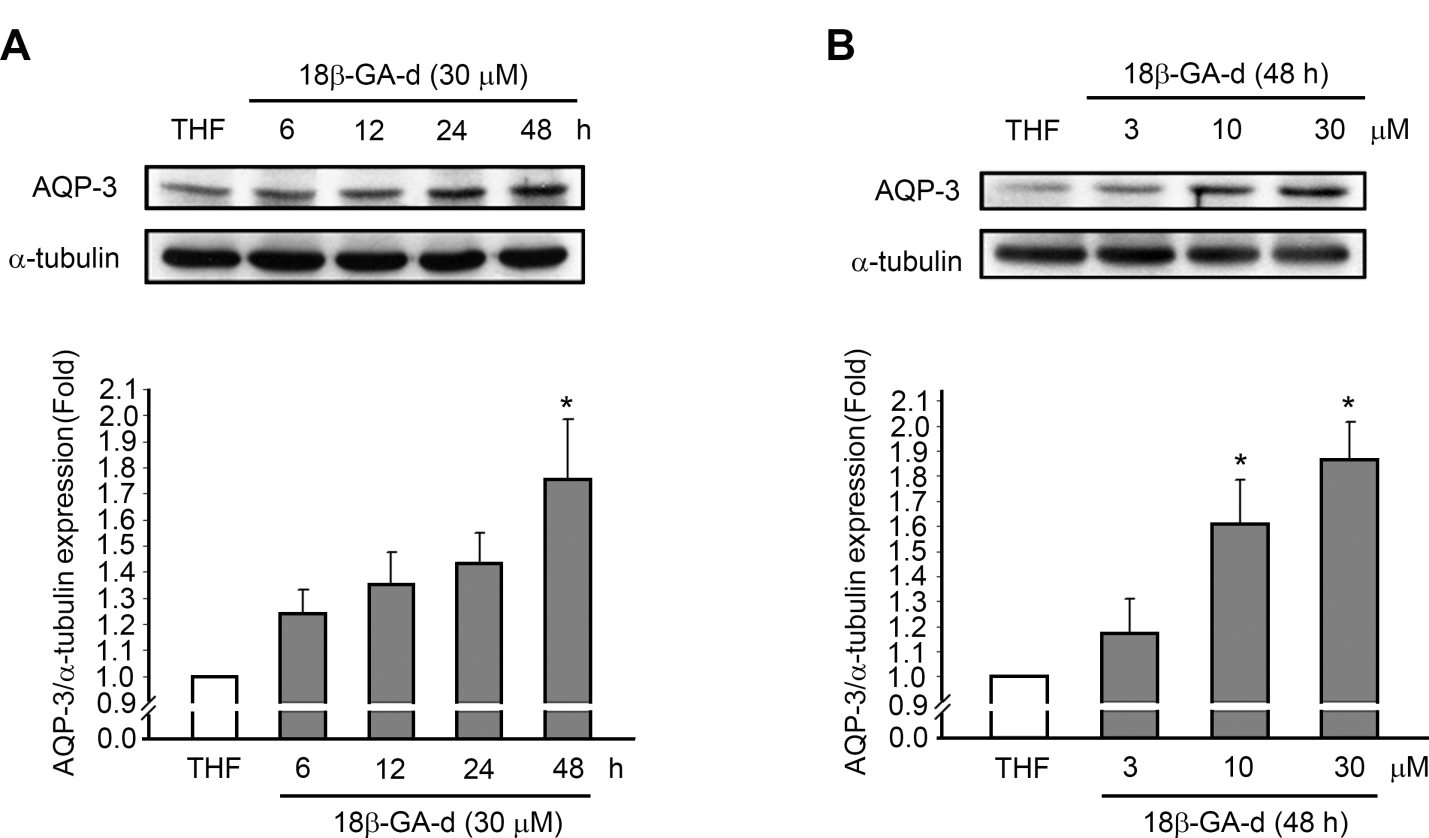
18β-GA-d induced AQP-3 expression in HaCaT keratinocytes. (A) HaCaT keratinocytes were treated with 0.1% THF or 18β-GA-d (30 μM) for different times (6, 12, 24, and 48 h). AQP-3 protein expression was analyzed by western blotting. (B) HaCaT keratinocytes were treated with 0.1% THF or different doses of 18β-GA-d (3, 10, and 30 μM) for 48 h. AQP-3 protein expression was analyzed by western blot assay. The quantification data for ratios of AQP-3/α-tubulin in 18β-GA-d-treated group, relative to AQP-3/α-tubulin in THF-treated group are shown in the lower panel. *p < 0.05 (mean ± S.E.M. n = 3).

As the 18β-GA-d was found to be capable of promoting the growth and migration of HaCaT keratinocytes, we investigated if it could induce the expression of AQP-3. We treated the HaCaT cells with 30 μM of the 18β-GA-d, and found that AQP-3 expression was induced (Fig 7A). However, the significant induction of AQP-3 in HaCaT keratinocytes was detected at later time, 48 h after treatment, than in human dermal fibroblasts. Regarding the effect of dosage, 10 and 30 μM of the 18β-GA-d induced the expression of AQP-3 in HaCaT keratinocytes, but 3 μM of the 18β-GA-d had no significant effects. These data suggested that 30 μM of the 18β-GA-d had similar pharmacological effects in keratinocytes as human dermal fibroblasts, although the effects on keratinocytes might be milder than that on the fibroblasts.

## Discussion

Both 18α-GA and 18β-GA isoforms are known to exhibit cytotoxic or suppressive effects on cell growth, and have been subjected to various cancer studies [6, 7]. It has been reported that 18α-GA could inhibit cell proliferation or induce apoptosis in breast and prostate cancer cell lines [31, 32]. It has also been shown to have prominent anti-cancer activities; it inhibits the growth of or induces apoptosis in different cancer cell lines. It has also been shown to regulate immunomodulation, both *in vitro* and *in vivo* [7]. Many synthesized derivatives of GA have also shown significant cytotoxic activities [7, 33]. The positives effects of 18α-GA, 18β-GA, and their derivatives on cell proliferation have not been extensively studied. In our study, the growth of dermal fibroblasts and epidermal keratinocytes was shown to be promoted by an 18β-GA-d, which had a distinct pharmacologic function. In contrast to other 18β-GA-ds, this compound significantly enhanced the growth of fibroblasts and keratinocytes: a remarkably unique effect. This compound also promoted the migration of fibroblasts and keratinocytes. Thus, in addition to developing 18β-GA-ds that could induce apoptosis, it is also important to develop non-cytotoxic 18β-GA derivatives such as the compound in this study and evaluate their possible pharmacologic roles in non-malignant diseases, including various skin disorders characterized by poor wound healing or atrophic skin lesions.

So far, most GA-ds have been synthesized based on the structure of 18β-GA, rather than that of the 18α stereo-isomeric form. The structural or chemical modifications of 18β-GA usually focus on the functional groups of ring A, C, E, or multiple rings [7, 8]. Many 18β-GA-ds show prominent cytotoxic activity [7, 33, 34], and most cytotoxic 18β-GA-ds showed optimum cytotoxicity at concentrations less than 30 μM [7]. The introduction of a double bond at C1-C2 in ring A, with an electronegative functional group at C2 and the oxidation of the hydroxyl group of C3 into a carbonyl group, has been shown to increase cytotoxicity [7]. The esterification of the C-30 carboxyl group has also been shown to change the polarity pattern and improve the antitumor activity [8]. In a previous study, we had investigated the anti-inflammatory and antioxidant activities of different GA-ds with structural modifications on the cleavage of ring A or introduction of various ester groups at C-3 and C-30. Many 18β-GA-ds, including the compound used in this study, have exhibited potency for inhibiting oxidative stress in neutrophils or tumor necrosis factor (TNF)-α formation in RAW 264.7 cells [9]. Further studies have to be performed to explore the ability of these 18β-GA-ds to prevent oxidative injuries in skin keratinocytes and fibroblasts, such as UVA and/or UVB-induced cellular damages [35, 36]. The unknown potential biological effects of other related compounds are being investigated.

In addition to the effects on cell growth and migration, the 18β-GA-d used in this study could also up-regulate expression of aquaporin-3 (AQP-3) in fibroblasts and keratinocytes. AQP-3 is critical for many essential physiological functions of the skin such as skin hydration and barrier recovery [20]. AQP-3 is also critically involved in epidermal proliferation and migration, and cutaneous wound healing has been shown to be impaired in AQP-3 knockout mice [22, 37]. In addition, AQP-3 plays an indispensable role in regulating the migration of skin dermal fibroblasts stimulated by the epidermal growth factor (EGF), suggesting its role in the normal wound healing process [23]. A recent study had demonstrated that treatment with topical erythropoietin could induce AQP-3 expression in the skin and promote the healing process of burn wounds in diabetic pigs through an AQP-3-dependent pathway [38]. These reports suggested that it is significant to develop promising novel pharmacological agents that can promote wound healing during diseased skin conditions by regulating AQP-3 expression in skin.

Regarding the regulation of AQP-3 expression, a previous report had shown that EGF could trigger the activation of EGFR, PI3K, and ERK signaling pathways to regulate the AQP-3 expression in dermal fibroblasts, which is involved in fibroblast migration [23]. In our study, the 18β-GA-d significantly up-regulated AQP-3 expression in dermal fibroblasts, and induced the phosphorylation of Akt, ERK, and p38. The inhibition of Akt by pharmacological inhibitors predominantly decreased the AQP-3 expression induced by the 18β-GA-d, suggesting that the Akt signaling pathways had important regulatory roles. To the best of our knowledge, very few licorice-related compounds can regulate the expression of AQP-3 [39–41], and other known 18β-GA-ds have never been reported to regulate AQP-3 expression in keratinocytes or fibroblasts, suggesting that this 18β-GA-d was unique. In addition, we investigated the effects of this 18β-GA-d on the expression of AQP-9 and AQP-10 in dermal fibroblasts, and found that the AQP-9 and AQP-10 mRNAs were also induced (data not shown). However, the known functions of AQP-9 and AQP-10 in skin fibroblasts are very limited and the significance should be further investigated.

The results of *in vitro* cellular experiments in this study demonstrated significant effects of the 18β-GA-d on the biological functions of primary human dermal fibroblasts; however, there are some limitations to this study. The *in vivo* functions of the 18β-GA-d are unknown and require further animal studies. Models of mouse skin diseases characterized by impaired wound healing, such as the diabetic wound model, or damaged epidermal keratinocytes and dermal fibroblasts, such as UVA/UVB-irradiation models, could be used for future studies [22, 25, 38]. In addition, the up-regulation of AQP-3 is known to be involved in proliferative skin lesions such as atopic dermatitis [42] or skin squamous cell carcinoma [43]. The potential effects of the AQP-3-inducing property of this compound on proliferative skin disorders are unknown and should be investigated.

In conclusion, we demonstrated that a synthesized 18β-GA-d was not toxic for skin cells, but could promote their migration, proliferation, and AQP-3 expression. The *in vitro* findings of this study would pave the way for further studies on the possible effects of this compound on diverse skin disorders.

## Supporting information

S1 Fig Original western blot images of Fig 4B. Images showing the expression of AQP-3 (upper panel) and α-tubulin (lower panel).
(JPG)

S2 Fig Original western blot images of Fig 7A. Images showing the expression of AQP-3 (upper panel) and α-tubulin (lower panel).
(JPG)

S3 Fig Original western blot images of Fig 7B. Images showing the expression of AQP-3 (upper and middle panels) and α-tubulin (lower panel).
(JPG)

## Abbreviations

18β-GA: 18β-glycyrrhetinic acid
18β-GA-d: 18β-glycyrrhetinic acid derivative
THF: Tetrahydrofuran, THF
AQP: Aquaporin
ERK: p42/44 extracellular signal regulated kinase
JNK: c-Jun NH2-terminal kinase
MTT: 3-(4,5-dimethylthiazol-2-yl)2,5-dephenyltetrazolium bromide
PMA: phorbol 12-myristate13-acetate
