# Supplementary figures and images for: 18ß-glycyrrhetinic acid derivative promotes proliferation, migration and aquaporin-3 expression in human dermal fibroblasts
Source: PLoS One. 2017 Aug 16;12(8):e0182981. doi: 10.1371/journal.pone.0182981 (PMC5558956; doi:10.1371/journal.pone.0182981)

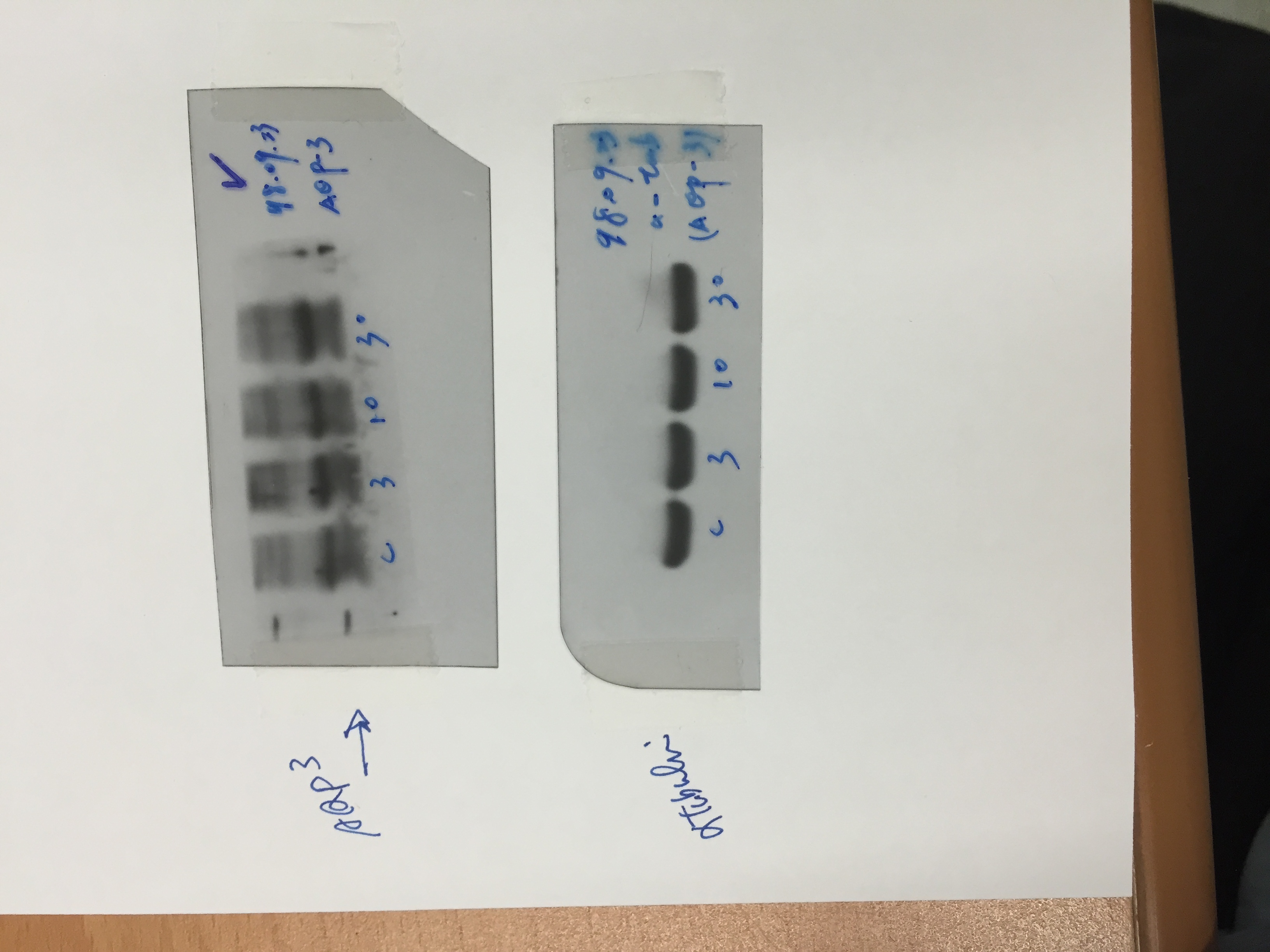

Supplement: S1 Fig — Images showing the expression of AQP-3 (upper panel) and α-tubulin (lower panel). (JPG) [file pone.0182981.s001.JPG]

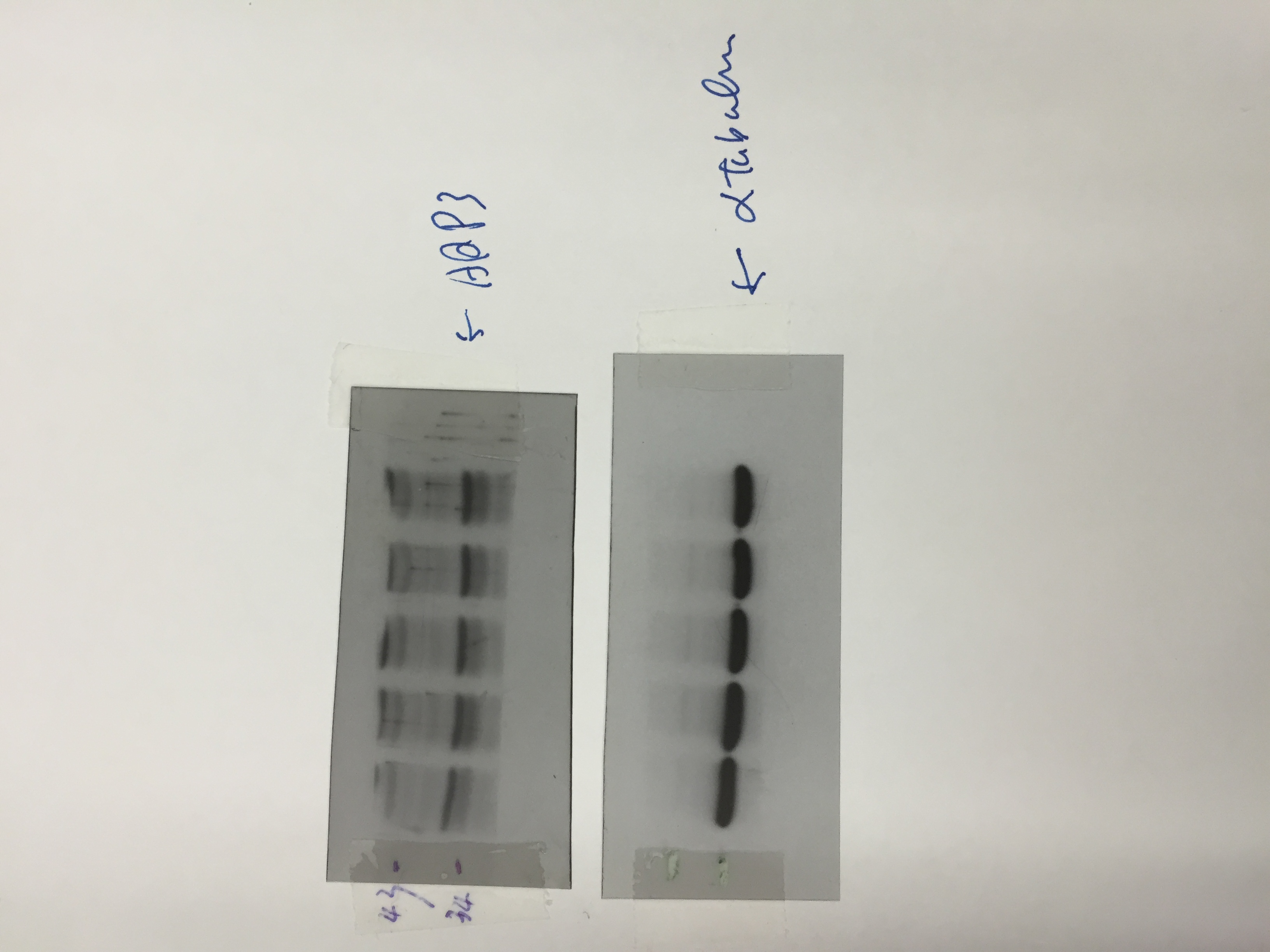

Supplement: S2 Fig — Images showing the expression of AQP-3 (upper panel) and α-tubulin (lower panel). (JPG) [file pone.0182981.s002.JPG]

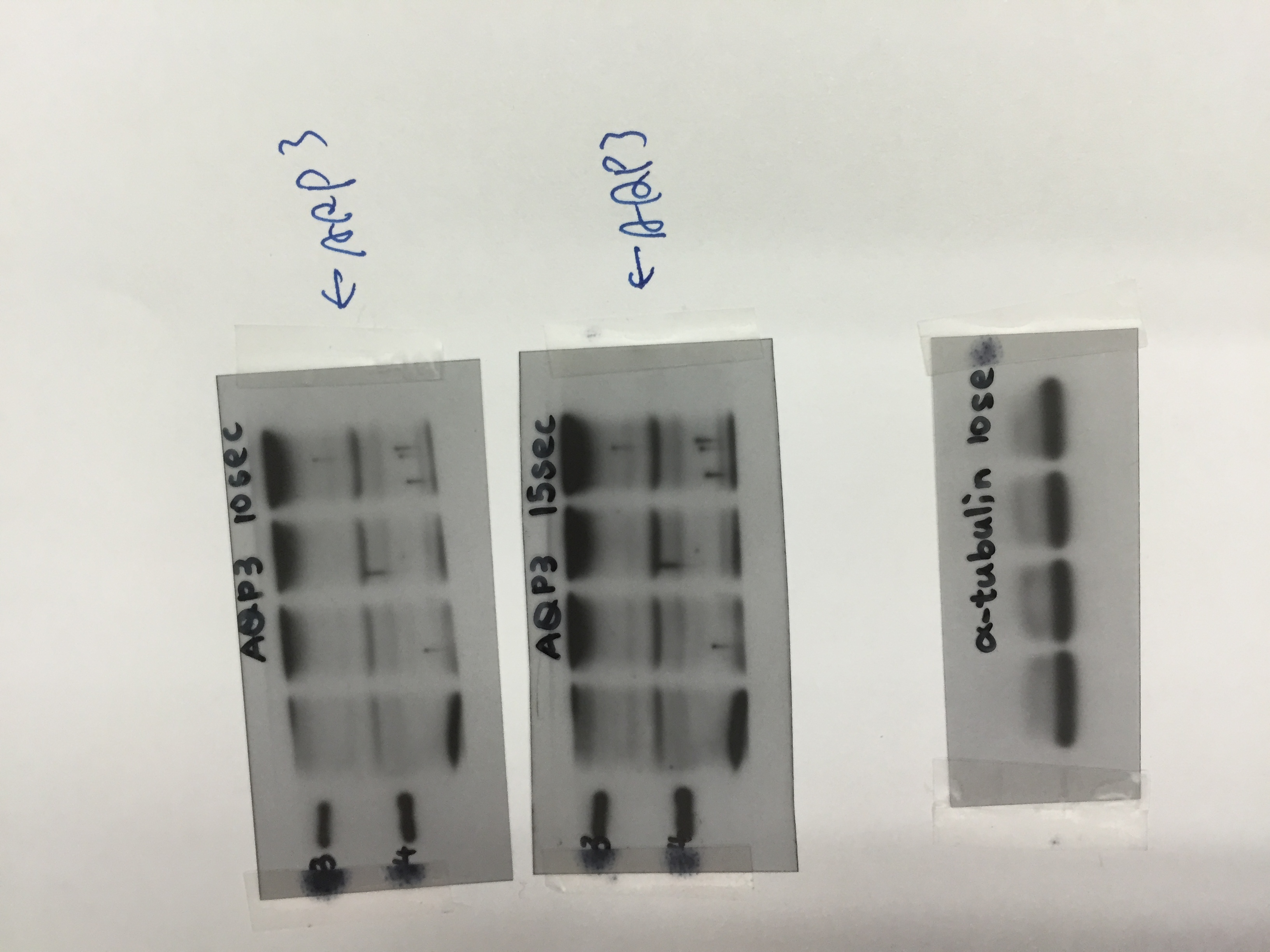

Supplement: S3 Fig — Images showing the expression of AQP-3 (upper and middle panels) and α-tubulin (lower panel). (JPG) [file pone.0182981.s003.JPG]
